# Brief Report: Hispanic Patients’ Trajectory of Cancer Symptom Burden, Depression, Anxiety, and Quality of Life

**DOI:** 10.3390/nursrep11020044

**Published:** 2021-06-09

**Authors:** Eida M. Castro-Figueroa, Normarie Torres-Blasco, Milagros C. Rosal, Julio C. Jiménez, Wallesca P. Castro-Rodríguez, Marilis González-Lorenzo, Héctor Vélez-Cortés, Alia Toro-Bahamonde, Rosario Costas-Muñiz, Guillermo N. Armaiz-Peña, Heather Jim

**Affiliations:** 1 Ponce Health Sciences University-Ponce Research Institute, Ponce 00716, Puerto Rico; normarietorres@psm.edu (N.T.-B.); jcjimenez@psm.edu (J.C.J.); marilisgonzalez@stu.psm.edu (M.G.-L.); hvelez@psm.edu (H.V.-C.); garmaiz@psm.edu (G.N.A.-P.); 2 Population and Quantitative Health Sciences, School of Medicine, University of Massachusetts Medical School, Worcester, MA 01605, USA; Milagros.Rosal@umassmed.edu; 3 Christiana Care Health Center-Helen F. Graham Care Center, Wilmington, DE 19899, USA; wallesca.p.castrorodriguez@coh.org; 4 Merck Pharmaceutical, Oncology Department, Kenilworth, NJ 07033, USA; alia.m.toro.bahamonde@merck.com; 5 Memorial Sloan Kettering Cancer Center, Department of Psychiatry and Behavioral Sciences, Puerto Rico, CA 00984, USA; costasmr@mskcc.org; 6 Moffitt Cancer Center, Tampa, FL 33612, USA; Heather.Jim@moffitt.org

**Keywords:** Cancer, Cancer Symptom Burden, Quality of Life, Depression, Anxiety, Hispanic/Latinx

## Abstract

**Background:** Anxiety and depression symptoms are known to increase cancer symptom burden, yet little is known about the longitudinal integrations of these among Hispanic/Latinx patients. The goal of this study was to explore the trajectory and longitudinal interactions among anxiety and depression, cancer symptom burden, and health-related quality of life in Hispanic/Latinx cancer patients undergoing chemotherapy. Methods: Baseline behavioral assessments were performed before starting chemotherapy. Follow-up behavioral assessments were performed at 3, 6, and 9 months after starting chemotherapy. Descriptive statistics, chi-square tests, Fisher’s exact tests, and Mann–Whitney tests explored associations among outcome variables. Adjusted multilevel mixed-effects linear regression models were also used to evaluate the association between HADS scores, follow-up visits, FACT—G scale, MDASI scale, and sociodemographic variables. Results: Increased cancer symptom burden was significantly related to changes in anxiety symptoms’ scores (adjusted $\hat{\beta }$ = 0.11 [95% CI: 0.02, 0.19]. Increased quality of life was significantly associated with decreased depression and anxiety symptoms (adjusted $\hat{\beta }$ = −0.33; 95% CI: −0.47, −0.18, and 0.38 adjusted $\hat{\beta }$= −0.38; 95% CI: −0.55, −0.20, respectively). Conclusions: Findings highlight the need to conduct periodic mental health screenings among cancer patients initiating cancer treatment.

## 1. Introduction

Anxiety and depression symptoms, cancer symptom burden, and health-related quality of life are common symptoms experienced by patients under cancer care [1,2,3], and there is a lack of effort within the scientific community to understand them among Hispanics/Latinos. For patients undergoing chemotherapy, depression symptoms are up to four times higher than the US general population [4]. Regarding Hispanic/Latino cancer patients living in the US, they are even more elevated [5,6]. A systematic review reported that Hispanic breast cancer patients were more likely to report poor mental, physical, and social quality of life [7], yet these are cross-sectional data. Anxiety, depression, and cancer symptom burden are known to predict cancer patients’ quality of life among non-Hispanic cancer patients [8,9]. Furthermore, cancer patients undergoing cancer care exhibit increased anxiety and depression symptoms at the time of diagnosis and initiation of their cancer treatment (Breen et al., 2009). Still, studies documenting this in Hispanics are very limited [10,11,12].

The aim and hypothesis of this study were conceptualized based on the Health-related Quality of Life conceptual model [13]. The aim was to explore the longitudinal relationship among anxiety and depression symptoms, cancer symptom burden, and health-related quality of life in Hispanic cancer patients. The team hypothesized that participants under active oncology treatment experiencing greater depressive and anxiety symptomatology will report higher levels of cancer symptom burden and lower health-related quality of life.

## 2. Materials and Methods

The study was approved by the Ponce Research Institute IRB committee (protocol #141007-EC). Baseline assessments were performed a week before starting chemotherapy, and quarterly for up to 9 months. Participants received a 10 USD incentive for each assessment/time point. 

### 2.1. Participants

Participant recruitment took place at a community oncology clinic located in an urban area of Puerto Rico. Participants were recruited by convenience during their follow-up oncology appointment. Considering that this was a pilot study, the team estimated the sample size on the availability of participants for recruitment during the proposed time frame. Participant eligibility: 21 years of age and over, confirmed cancer diagnosis and scheduled to receive the first course of chemotherapy within the next 2 weeks after baseline assessment. Participant exclusion: had received any other cancer therapy (e.g., radiotherapy, hormonal therapy) 5 years prior to the recruitment date; were experiencing a depressive mood at the time of screening; use of illicit drugs or alcohol use disorder; history of psychotic disorders. A total of 96 patients were screened and 52 met the inclusion criteria. 

### 2.2. Measures

Patient socio-demographic and clinical data: socio-demographic information was collected using a self-reported measure. Socio-demographic variables included age, sex, monthly income, education level, marital status, employment status, medical insurance, as well as religious and spiritual practices. Clinical information was gathered through medical chart reviews and included tumor site, tumor stage, and type of treatment. Self-reported personal and family history of mental health disorders data were collected.

Anxiety and depression symptoms: symptoms of anxiety and depression were assessed using the Spanish version of the Hospital and Anxiety Depression Scale (HADS). This 14-item scale evaluates the frequency of experiencing anxiety and depression symptoms. The HADS total scale Cronbach alpha is 0.88, 0.85 for the anxiety sub-scale, and 0.83 for the depression sub-scale [14]. It includes somatic symptoms of anxiety and depression (headaches, fatigue, insomnia, anergia, etc.) that can be caused by side effects related to cancer and its treatment [15]. 

Disease- and treatment-related symptoms: physical symptoms were assessed using the MD Anderson Symptom Inventory (MDASI). The MDASI [16] is a 19-item scale that measures cancer symptom severity (Cronbach alpha = 0.91) and its interference (Cronbach alpha = 0.85) with daily life activities. Symptoms at their worst over the past 24 h and interference with daily activities are rated by participants on a 0 to 10 Likert scale. 

Health quality of life: health-related quality of life was evaluated with the Spanish version of the Functional Assessment of Cancer Therapy—General Version 4 (FACT—G) developed by Cella [17]. This is a 28-item scale designed to rate physical, social/family, emotional, and functional well-being using a 5-point Likert scale. Test–retest correlation reliability coefficients were 0.92 for the total score, 0.88 for physical well-being, 0.84 for functional well-being, 0.82 for social well-being, and 0. 82 for emotional well-being.

### 2.3. Data Analysis

Descriptive statistics were used to characterize participants in the study. The association between depressive symptoms and socio-demographic characteristics was calculated using chi-square tests, Fisher’s exact tests, and Mann–Whitney tests, as appropriate. Additionally, adjusted multilevel mixed-effects linear regression models were used to classify the association between HADS scores for depression and anxiety, period of time receiving oncology treatment (follow-up visits), participants’ quality of life (FACT—G scale), cancer symptoms burden (MDASI scale), age, sex, and marital status; all variables were included in the adjusted model. A total of 50 participants were included in the linear mixed models, which used the full data set despite missing values at some time points. Data were missing at random, hence the means obtained from these models could be considered unbiased estimates. P-values less than 0.05 were considered statistically significant. Stata v.14 [18] was used to analyze all the data. 

## 3. Results

**Figure 1:**
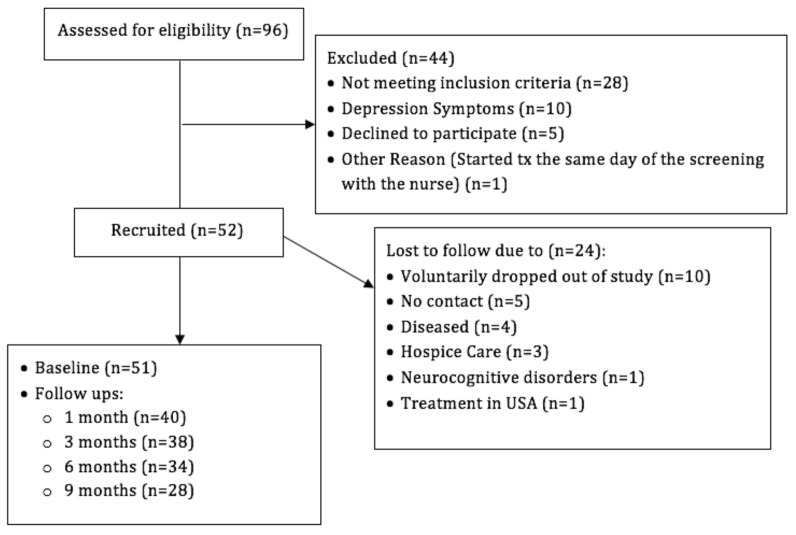
Presents patients’ recruitment retention data and the reasons for not meeting the inclusion criteria. The recruitment rate was 60.46%, and the attrition rate was 56%. Screened patients (n = 10) and participants enrolled in the study (n = 5) who met clinical criteria for clinically significant depression/anxiety symptoms were referred to mental health services.

Figure 1 presents patients’ recruitment retention data and the reasons for not meeting the inclusion criteria. The recruitment rate was 60.46%, and the attrition rate was 56%.

### 3.1. Participants’ Socio-Demographic and Clinical Characteristics

More than half of this study’s participants were males (58.8%), had high school or greater education (70.6%), and were married (62.8%). Participants’ mean age was 63.3 (±14.6) years. The distribution of other socio-demographic and clinical characteristics is shown in Table 1. The most common cancer types among participants included colon and rectum (19.6%), breast (11.8%), and pancreas (11.8%); most of the malignancies were diagnosed in an advanced stage (44%). About 39.3% (11 out of 28 who completed all study measures) of the participants exhibited clinically significant depressive symptoms at least once during the study period. No associations were found between the presence of depressive symptoms and socio-demographic characteristics (*p* > 0.05; Table 2).

### 3.2. Correlations among Anxiety, Depression, Cancer Symptom Burden, and Quality of Life

Overall, a slight reduction in anxiety (95% CI: −0.28, 0.18) and depression (95% CI: −0.12, 0.14) symptoms was observed through time among the participants, although not statistically significant. After adjusting for sex, age, marital status, quality of life, burden of cancer symptoms, and repeated measures effect among each participant, the overall HADS score mean during baseline measurements was 7.97 (95% CI: 4.43, 11.51) for depression and 11.06 (95% CI: 6.76, 15.36) for anxiety. The adjusted difference of the HADS score mean indicates that, between visits, participants had lower scores of depression symptoms during the first (1 month) and fourth (9 months) follow-up assessments as compared to baseline scores; changes in scores through time were not statistically significant. The HADS score for anxiety symptoms showed a significant decrease from 11.06 at baseline to 9.83 by the fourth visit (9 months) (adjusted $\hat{\beta }$ = −1.23, 95% CI: −2.27, −0.19). 

### 3.3. Hypothesis Testing Multilevel Mixed-Effects Linear Regression Results

Table 3 illustrates associations between quality of life, depression, and anxiety symptoms, accordingly. Each 5-unit increase in the FACT—G scale (i.e., better quality of life) was associated with significantly decreased HADS scores of depression and anxiety (adjusted $\hat{\beta }$ = −0.33; 95% CI: −0.47, −0.18) and 0.38 (adjusted $\hat{\beta }$ = −0.38; 95% CI: −0.55, −0.20), individually, once the team adjusted for confounders such as sex, age, and marital status. Meanwhile, another 5-unit increase in the burden of cancer symptoms showed significant changes in the HADS scores for anxiety (adjusted $\hat{\beta }$ = 0.11 [95% CI: 0.02, 0.19]; Table 3). Sub-scales for symptom intensity and interference of symptoms with different aspects of the participants’ life showed median values of 19 (Interquartile Range [IQR] = 5, 31) and 6 (IQR = 1.3, 19.3), correspondingly. Furthermore, higher HADS scores of anxiety were observed among women as compared to men (adjusted $\hat{\beta }$ = 1.91; 95% CI: 0.58, 3.24). 

Random effects for depression (LR test vs. linear model = 34.06; *p* < 0.0001) and anxiety (LR test vs. linear model = 21.72; *p* < 0.0001) showed that, for any visit, each participant might have a distinct intercept HADS scores up to 3.22 (depression) and 3.79 (anxiety) higher or lower than the average obtained from all participants as a group about 95% of the time. The variability of individual HADS scores during visits is around 1.60 and 2.00 for depression and anxiety, respectively, around the individual regression lines for each participant. Approximately 50.4% and 47.6% of the variance in HADS scores for depression and anxiety, respectively, can be attributed to differences between participants.

## 4. Discussion

At baseline, HADS scores were higher for anxiety symptoms than depression and, although anxiety symptoms decreased over time, these remained clinically significant. Rates of anxiety and depression after cancer diagnosis and before initiating oncology treatment may differ by type of cancer [19,20,21]. For instance, some studies report higher depression symptoms after cancer diagnosis and around the time of starting oncology treatment [22,23]. Symptoms of anxiety and depression diminished over time, with changes in depression symptoms being marginally significant. A study of colorectal cancer patients found that depression symptoms changed over time, where a longer time since diagnosis was associated with fewer depressive symptoms [24]. Even so, no significant changes over time in anxiety symptoms were found [24]. Another study revealed that clinical symptoms of anxiety and depression were prevalent at the completion of chemotherapy and, after completion of chemotherapy, depression symptoms decreased, and anxiety symptoms increased [25]. Another study found that symptoms of depression increased from pre-surgery up to 6 months, and symptoms of anxiety remained stable [26]. Similar to our results, Vahdaninia and colleagues found that anxiety and depression symptoms also improved over time [27].

Finally, as expected, the pilot cohort study found significant associations among anxiety, depression, and quality of life. Yet, no significant associations were found between changes in anxiety and depression and cancer symptom severity throughout 9 months. One potential explanation for the differences in the findings and results from other research groups could be that the team excluded patients that reported depression symptoms at the time of recruitment (exclusion criteria #2). The team excluded these patients because they wanted to observe the course of depression symptoms that emerged after cancer treatment. Thus, the interpretation of these results may apply exclusively to cancer patients who do not present clinically significant depression symptoms at the time of cancer treatment initiation. Of note, other studies have yielded significant associations among symptoms of depression, anxiety, and cancer symptom burden. For instance, a study conducted by Shi and collaborators revealed that, among cancer patients reporting high cancer symptom burden, depression (along with fatigue and pain) had the most significant impact on their quality of life [28]. Two cross-sectional studies among patients with advanced-staged cancer found significant correlations between depression severity, number of reported physical symptoms, and symptom severity independent of cancer type [29,30]. Another study among breast cancer patients that utilized the Distress Thermometer’s Physical Problem List revealed that most physical problems were associated with depression (87% of symptoms) and anxiety (53% of symptoms) [31]. Likewise, Leonhart and colleagues found that, among other factors, anxiety and depression symptoms predicted higher somatic symptom severity [32,33].

## 5. Conclusions

This pilot study suggests cancer patients experience higher symptoms of anxiety at chemotherapy treatment initiation. Moreover, even though anxiety symptoms decreased over time, such symptoms remained high. These findings highlight the need to conduct periodic mental health screenings among cancer patients initiating cancer treatment. This study will inform a large-sample observational longitudinal study aimed at developing a comprehensive model predicting the longitudinal mediation and moderator interactions among anxiety and depression symptoms, cancer symptom burden, and health-related quality of life. Such study is needed to inform effective mental health screening efforts and interventions targeting Hispanic cancer patients under active oncology treatment. 

## Figures and Tables

**Table 1:** Socio-demographics and clinical features of participants (n = 51).

Socio-Demographics	
**Age**	
Mean ± SD	63.3 ± 14.6
Median (min, max)	66 (23, 86)
**Sex, n (%)**	
Male	30 (58.8)
Female	21 (41.2)
**Household composition, n (%)**	
Alone	8 (15.7)
Partner	19 (37.3)
Son/Daughter	5 (9.8)
Parents	2 (3.9)
Partner and Son/Daughter	10 (19.6)
Parents and Son/Daughter	2 (3.9)
Son/Daughter and Grandchildren	2 (3.9)
Partner, Son/Daughter, and Grandchildren	1 (2.0)
Other Family Members	2 (3.9)
**Employment, n (%)**	
Employed	6 (11.8)
Unemployed	8 (15.7)
Disabled	4 (7.8)
Retired	30 (58.8)
Student	1 (2.0)
Other (did not specify)	2 (3.9)
**Education, n (%)**	
<High School	15 (29.4)
≥High School	36 (70.6)
**Civil Status, n (%)**	
Single	7 (13.7)
Married/Living with Partner	32 (62.8)
Divorced	6 (11.8)
Widowed	6 (11.8)
**Medical Insurance ^†^, n (%)**	
Private ^‡^	14 (28.0)
Health Care Reform ^§^	14 (28.0)
Medicare	22 (44.0)
**Household Income, n (%)**	
≤$19,000	37 (72.6)
>$19,000	14 (27.4)
**Clinical Features**	
**Tumour site (primary), n (%)**	
Breast	6 (11.8)
Prostate ^††^	5 (9.8)
Multiple Myeloma	2 (3.9)
Head and Neck	3 (5.9)
Leukaemia	2 (3.9)
Lung	3 (5.9)
Pancreatic	6 (11.8)
Lymphoma	5 (9.8)
Colorectal	10 (19.6)
Stomach	2 (3.9)
Melanoma	2 (3.9)
Other sites ^‡‡^	5 (9.8)
**Disease stage, n (%)**	
I ^††^	6 (12.0)
II	9 (18.0)
III	13 (26.0)
IV	22 (44.0)

^†^ One missing value. ^‡^ Four participants had additional medical insurance; three with health care reform and one with Medicare. ^§^ Two participants also had Medicare. ^††^ One patient also had a stage III multiple myeloma. ^‡‡^ Includes Hodgkin, renal, urinary, gynaecological and mantel cell malignancies.

**Table 2:** Association between socio-demographic characteristics and presence of depressive symptoms (n = 28).

Characteristics	Without Depressive Symptoms (n = 17)	With Depressive Symptoms (n = 11)	*p*-Value ^†^
**Sex**			0.14 ^‡^
Male	11 (64.7)	4 (36.4)	
Female	6 (35.3)	7 (63.6)	
**Age**			0.72
Mean (+/− SD)	59.2 (14.9)	62.4 (15.5)	
Median (P25–P75)	65 (55–67)	63 (47–78)	
**Household Income**			0.08
≤$19,000	15 (88.2)	6 (54.6)	
>$19,000	2 (11.8)	5 (45.5)	
**Income-Enough**			0.14 ^‡^
No	6 (35.3)	7 (63.6)	
Yes	11 (64.7)	4 (36.4)	
**Education**			0.65
<High School	3 (17.7)	3 (27.3)	
≥High School	14 (82.4)	8 (72.7)	
**Marital status**			0.08
Single	4 (23.5)	0 (0.0)	
Married/Living with a partner	9 (52.9)	10 (90.1)	
Divorced	3 (17.7)	0 (0.0)	
Widowed	1 (5.9)	1 (9.1)	

Individuals who did not present depressive symptoms and did not complete all study visits were not included in the analysis due to possible misclassification as non-depressive symptoms. ^†^ *p*-value was calculated using Fisher’s Exact test and Mann-Whitney test when appropriate unless otherwise specified. ^‡^ *p*-value was calculated using Chi-square test.

**Table 3:** Mixed linear regression models of HAD scale scores for depression and anxiety among individuals with cancer diagnosis receiving treatment (n = 51).

Fixed Effect	Depression Symptoms $Estimated\;\beta \;\left(95\%CI\right)$	Anxiety Symptoms $Estimated\;\beta \;\left(95\%CI\right)$
**Intercept**	7.97 (4.43, 11.51)	11.06 (6.76, 15.36)
**Time point (visits)**		
Baseline	REFERENCE	REFERENCE
1	−0.23 (−0.94, 0.48)	−0.72 (−1.60, 0.16)
2	0.19 (−0.54, 0.92)	−0.19 (−1.10, 0.71)
3	−0.00 (−0.75, 0.75)	−0.54 (−1.47, 0.39)
4	−0.39 (−1.23, 0.45)	−1.23 (−2.27, −0.19) ^‡^
**Sex**		
Male	REFERENCE	REFERENCE
Female	0.85 (−0.26, 1.96)	1.91 (0.58, 3.24) ^‡^
**Age** ^†^	−0.12 (−0.33, 0.10)	−0.24 (−0.50, 0.02)
**Marital Status**		
Single	REFERENCE	REFERENCE
Married/Living with Partner	1.57 (−0.24, 3.38)	1.00 (−1.15, 3.16)
Divorced	0.29 (−1.95, 2.54)	−0.37 (−3.05, 2.31)
Widowed	0.95 (−1.74, 3.65)	1.80 (−1.43, 5.03)
**Quality of Life** ^†^	−0.33 (−0.47, −0.18) ^‡^	−0.38 (−0.55, −0.20) ^‡^
**Burden of Cancer Symptoms** ^†^	0.04 (−0.03, 0.11)	0.11 (0.02, 0.19) ^‡^

^†^ Results are based on five units increase in the variable. ^‡^ *p*-value < 0.05.

## Data Availability

The data presented in this study will be available in the supplementary material contained in this article.
